# Risk factors for excessive gestational weight gain in a UK population: a biopsychosocial model approach

**DOI:** 10.1186/s12884-020-03519-1

**Published:** 2021-01-10

**Authors:** S. M. Garay, L. A. Sumption, R. M. Pearson, R. M. John

**Affiliations:** 1 Biomedicine Division, School of Biosciences, Cardiff University, Cardiff, CF10 3AX UK; 2 Centre for Academic Mental Health, Population Health Sciences, Bristol Medical School, University of Bristol, Oakfield House, Bristol, BS8 2BN UK

## Abstract

**Background:**

Gestational weight gain (GWG) can have implications for the health of both mother and child. However, the contributing factors remain unclear. Despite the advantages of using a biopsychosocial approach, this approach has not been applied to study GWG in the UK. This study aimed to investigate the risk factors of excessive GWG in a UK population, employing a biopsychosocial model.

**Methods:**

This study utilised data from the longitudinal Grown in Wales (GiW) cohort, which recruited women in late pregnancy in South Wales. Specifically, data was collected from midwife recorded notes and an extensive questionnaire completed prior to an elective caesarean section (ELCS) delivery. GWG was categorised according to Institute of Medicine (IOM) guidelines. The analysis was undertaken for 275 participants.

**Results:**

In this population 56.0% of women had excessive GWG. Increased prenatal depression symptoms (Exp(B)=1.10, *p*=.019) and an overweight (Exp(B)=4.16, *p*<.001) or obese (Exp(B)=4.20, *p*=.010) pre-pregnancy BMI, consuming alcohol in pregnancy (Exp(B)=.37, *p*=.005) and an income of less than £18,000 (Exp(B)=.24, *p*=.043) and £25–43,000 (Exp(B)=.25, *p*=.002) were associated with excessive GWG.

**Conclusion:**

GWG is complex and influenced by a range of biopsychosocial factors, with the high prevalence of excessive weight gain in this population a cause for concern. Women in the UK may benefit from a revised approach toward GWG within the National Health Service (NHS), such as tracking weight gain throughout pregnancy. Additionally, this research provides evidence for potential targets for future interventions, and potentially at-risk populations to target, to improve GWG outcomes.

**Supplementary Information:**

The online version contains supplementary material available at 10.1186/s12884-020-03519-1.

## Background

The weight a woman gains during pregnancy, or gestational weight gain (GWG), can have implications for the health of both mother and child [1–3]. Inadequate GWG has been associated with higher risk of small-for-gestational age (SGA) infants and preterm birth [4]. Conversely, excessive GWG is suggested to be related to higher risk of large-for-gestational age infants (LGA), macrosomia, caesarean section (CS) delivery [4, 5], postpartum weight retention [6], gestational hypertension and augmentation of labour [7]. The timing of the excessive GWG in pregnancy could also be of importance, with the suggestion that there may be a critical period where GWG is most detrimental [3]. Additionally, there is recent evidence suggesting GWG is associated with childhood obesity [3, 8, 9].

In 2009 the Institute of Medicine updated their existing guidelines on recommended GWG to incorporate the World Health Organisation (WHO) maternal pre-pregnancy body mass index (BMI) categories. These guidelines advise underweight women to gain 15.5-18 kg, healthy-weight women 11.5-16 kg, overweight women 7–11.5 kg and obese women 5-9 kg [10]. However, a recent systematic review of over one million pregnant women demonstrated that only 30% of women obtained the recommended GWG, with 23 and 47% having inadequate or excessive GWG, respectively [4]. It has been suggested that the prevalence of excessive GWG is increasing [11].

Pre-pregnancy overweight or obese BMI has consistently been identified as a risk factor for excessive GWG across a range of countries [12, 13]. However, evidence for other potential contributing factors is mixed. Studies have suggested a variety of contributing factors including lower socioeconomic status or social inequalities [3, 13], increased food intake and height [12], an age of over 30 years [14], hypertension [15] and parity [16]. Existing literature does not consistently employ a biopsychosocial approach, which explicitly recognises the individual and important interacting influences of biomedical, psychological and social factors on health, despite the growing consensus that this may help explain the complex nature of GWG [12]. We were unable to identify previous research examining the prevalence and risk factors of GWG in the United Kingdom (UK). This is an important oversight as, unlike other countries such as America, in the UK GWG is not tracked through pregnancy [17]. It has been reported that, in the UK pregnant women were generally unconcerned about GWG, with the suggestion that this was partly due to a lack of information from health professionals who were unsure of what to advise regarding GWG [18]. The National Institute for Health and Care Excellence (NICE) recommends that all pregnant women, in particular women with a high BMI, receive guidance on diet and physical activity but there is no specific emphasis on weight gain.

The aim of the current study was to investigate the potential biopsychosocial risk factors for excessive GWG in a UK population, utilising the Grown in Wales (GiW) cohort.

## Method

### Participants

The Grown in Wales cohort is a longitudinal study in the South Wales region of the United Kingdom, which has previously been described in detail [19]. Briefly, women with a term pregnancy were recruited by research midwives at the University Hospital of Wales, at the presurgical appointment for an elective caesarean section (ELCS) between 1st September 2015 and 31st November 2016. Women were invited to participate in the study if it was a singleton term pregnancy without infectious diseases or fetal anomalies. Full ethical approval was obtained via the Wales Research Ethics Committee (REC), reference 15/WA/0004.

Initially 355 women were recruited to the GiW cohort, with seven later withdrawing. The current study focused on participants who were at 37 weeks gestation or above. Participants were excluded if there was no available data on gestational weight gain, either due to missing pre-pregnancy BMI or delivery weight. This left 275 participants for the current analysis.

### Materials

The current analysis utilised data collected from midwife recorded medical notes and an extensive questionnaire (Supplementary file 1) completed at the presurgical appointment prior to the booked ELCS.

#### Lifestyle

Data on lifestyle was obtained from the questionnaire. Lifestyle variables included in the analysis were exercise (defined as exercise for at least 30 min, at least once a week), smoking (tobacco), alcohol intake and dietary patterns during pregnancy. The specific dietary patterns were Western and Health Conscious, with the method of obtaining these dietary patterns previously outlined in detail [20].

#### Biological

Biological variables including maternal age, parity, pre-pregnancy BMI and fetal sex were obtained from the questionnaire and midwife recorded notes.

#### Psychological/mental health

The questionnaire incorporated a measure of depression symptoms, using the Edinburgh Postnatal Depression Scale (EPDS) [21], and a measure of trait anxiety, via the trait subscale of the Spielberger State-Trait Anxiety Inventory (STAI) [22], both of which have been validated for use in the perinatal period [23, 24]. The EPDS is a 10-item questionnaire that reflects how a person has felt in the previous 7 days, with responses selected from a 4-point scale. There is a maximum possible score of 30, with a score ≥ 13 indicative of probable depression [21, 25]. A review reported sensitivity and specificity estimates in the range of 64–100% and 73–100% respectively [23]. In the current study Cronbach’s Alpha for the EPDS was .86. The trait subscale of the STAI is a 20-item questionnaire that assesses anxiety levels in general, with sensitivity and specificity estimates of 80.95% and 79.75% respectively [26]. Items are rated on a 4-point scale (i.e. from “Almost never” to “Almost always”), with a maximum possible score of 80 and a score of ≥ 40 recommended as indicative of high anxiety [27]. In our study, the Cronbach’s Alpha for the STAI was found to be .91.

#### Sociodemographic

Sociodemographic information was obtained from the questionnaire and included data on ethnicity, income and education levels. Welsh Index of Multiple Deprivation (WIMD) 2014 scores were calculated from anonymised postcodes (http://wimd.wales.gov.uk). WIMD scores have a possible range of 0 to 1909, with a lower score indicative of living in an area of higher deprivation and conversely a high score indicative of a lower deprivation area.

#### GWG

GWG was calculated by the researchers utilising the self-reported pre-pregnancy weight from the questionnaire and weight at delivery recorded by the research midwife. Categories within GWG were determined utilising the Institute of Medicine (IOM) recommendations for weight gain during pregnancy [10].

### Statistical analysis

All analyses were undertaken utilising IBM SPSS Statistics Version 25. Normality was assessed, with all appropriate variables identified as non-parametric. Kruskal-Wallis H test was employed to assess any differences in GWG across the various indications for ELCS delivery. Frequencies of participants in each IOM category were produced, both overall and split by pre-pregnancy BMI. Risk factors for excessive compared to normal GWG were assessed utilising binary logistic regression, performed utilising all potential biopsychosocial variables. Reference categories were determined by selecting the category with the largest frequency. Given the biopsychosocial nature of this research, all variables were entered simultaneously in each analysis. The binary logistic regression was then adjusted to include only those potential variables significant at *p* < .15 in the final models, similar to that utilised in existing literature [12]. Multicollinearity was assessed and found not to be present thus all identified variables were included in the regression models.

## Results

Descriptive statistics for the participants eligible for inclusion in this analysis can be found in Table 1. Of these 275 participants, 42 (15.3%) were in the category of inadequate GWG, 79 (28.7%) normal GWG and 154 (56.0%) in the excessive GWG category. The median GWG was 14.86 kg (IQR = 7.90). Frequencies of participants in each IOM category when split by pre-pregnancy BMI can be found in Table 2. The median weight gain of participants with an underweight pre-pregnancy BMI was 17.85 kg (IQR = 23.37, range = 60.6 kg), a healthy pre-pregnancy BMI was 15.42 kg (IQR = 7.29, range = 59.7 kg), an overweight pre-pregnancy BMI was 15.60 kg (IQR = 7.70, range = 27.4 kg) and an obese pre-pregnancy BMI was 10.20 kg (IQR = 10.81, range = 53.0 kg). All women delivered by ELCS and differences in GWG between indications for ELCS, listed in Table 1, were assessed. There was no significant difference in GWG between indications (*p* = .240).

**Table 1:** Demographics for the 275 participants for whom gestational weight gain data were available

Demographics	% (n) or median (IQR)
Fetal sex *% (n)*	
Female	55.3 (152)
Male	44.7 (123)
Gestational age (weeks)	39.0 (.0)
BMI pre-pregnancy *% (n)*	
Underweight	2.2 (6)
Normal	51.6 (142)
Overweight	30.2 (83)
Obese	16.0 (44)
Maternal age at booking	34.0 (7.0)
Maternal ethnicity *% (n)*	
Caucasian	92.4 (254)
Other	7.6 (21)
Parity *% (n)*	
Multiparous	79.6 (219)
Nulliparous	20.4 (56)
Indications for ELCS *% (n)*	
Previous caesarean section	55.9 (147)
Previous pregnancy complication	15.6 (41)
Current pregnancy complication	20.5 (54)
Maternal choice	3.4 (9)
Maternal disorder (non-pregnancy related)	4.6 (12)
Highest education level *% (n)*	
Left before GCSE	5.9 (16)
GCSE & Vocational	19.9 (54)
A-level	11.4 (31)
University	33.9 (92)
Postgraduate	28.8 (78)
Family income (£) *% (n)*	
< 18,000	7.5 (20)
18–25,000	8.6 (23)
25–43,000	19.4 (52)
> 43,000	52.2 (140)
Do not wish to say	12.3 (33)
WIMD score	1298.0 (1230.0)
Smoking in pregnancy^a^ *% (n)*	
No	90.1 (246)
Yes	9.9 (27)
Alcohol in pregnancy^a^ *% (n)*	
No	62.6 (169)
Yes	37.4 (101)
Exercise *% (n)*	
No	84.6 (231)
Yes	15.4 (42)
Western dietary pattern	−.04 (1.35)
Health conscious dietary pattern	.05 (1.52)
Term EPDS score	7.0 (6.0)
Term STAI score	34.0 (12.0)

*BMI* Body Mass Index, *WIMD score* Welsh Index of Multiple Deprivation score, *EPDS* Edinburgh Postnatal Depression Scale, *STAI* State-Trait Anxiety Inventory, *GCSE* General Certificate of Secondary Education, *A level* Advanced Level. ^a^ At any point in pregnancy

**Table 2:** Frequencies of participants in each IOM category when separated by pre-pregnancy BMI

BMI pre-pregnancy	IOM category	% (*n*)
Underweight	Inadequate	33.3 (2)
	Normal	16.7 (1)
	Excessive	50.0 (3)
Normal	Inadequate	15.5 (22)
	Normal	39.4 (56)
	Excessive	45.1 (64)
Overweight	Inadequate	4.8 (4)
	Normal	19.3 (16)
	Excessive	75.9 (63)
Obese	Inadequate	31.8 (14)
	Normal	13.6 (6)
	Excessive	54.5 (24)

Analysis of potential risk factors of excessive compared to normal GWG was undertaken utilising multivariate binary logistic regression (Table 3). Variables with the strongest associations at the unadjusted multivariate level were considered for the final analysis. After assessing for multicollinearity, all potential variables were included in the final adjusted multivariate binary logistic regression (Table 4). In this analysis, an increase in EPDS total score of 1 was associated with increased odds of excessive compared to normal GWG, by a factor of 1.10. Having an overweight or obese BMI pre-pregnancy compared to normal was associated with increased odds of excessive compared to normal GWG by a factor of 4.16 and 4.20, respectively. Consuming alcohol in pregnancy was associated with decreased odds of excessive GWG by a factor of .37. A family income of less than £18,000 or £25–43,000 compared to greater than £43,000 was associated with decreased odds of excessive GWG by a factor of .24 and .25, respectively.

**Table 3:** Unadjusted multivariate binary logistic regression identifying significant risk factors of excessive compared to normal GWG

	Excessive vs normal GWG		
	P	Exp(B)	95% CI
Fetal sex			
Female	*ref*		
Male	.575	.79	.36, 1.78
Gestational age (weeks)	.431	1.27	.70, 2.31
BMI pre-pregnancy			
Underweight	1.000	.00	.00, .00
Normal	*ref*		
Overweight	**<.001**	4.78	2.01, 11.34
Obese	**.004**	8.76	2.00, 38.39
Maternal age at booking	.170	1.07	.97, 1.19
Parity			
Multiparous	*ref*		
Nulliparous	.266	1.77	.65, 4.85
Highest education level			
Left before GCSE	.262	.29	.04, 2.49
GCSE & Vocational	.746	1.21	.38, 3.86
A-level	.132	3.25	.70, 15.12
University	*ref*		
Postgraduate	.832	.91	.37, 2.24
Family income			
< 18,000	.264	.34	.05, 2.27
18–25,000	.332	2.66	.37, 19.19
25–43,000	.125	.42	.14, 1.27
> 43,000	*ref*		
Do not wish to say	.360	.53	.13, 2.08
WIMD score	.802	1.00	1.00, 1.00
Smoking in pregnancy^a^			
No	*Ref*		
Yes	.231	.37	.07, 1.89
Alcohol in pregnancy^a^			
No	*Ref*		
Yes	**.007**	.33	.15, .74
Exercise			
No	*Ref*		
Yes	.621	1.28	.49, 3.34
Western dietary pattern	.125	1.40	.91, 2.16
Health conscious dietary pattern	.475	1.19	.74, 1.89
Term EPDS score	**.033**	1.15	1.01, 1.31
Term STAI score	.229	.96	.89, 1.03

*CBWC* Custom birthweight centiles, *BMI* Body Mass Index, *WIMD score* Welsh Index of Multiple Deprivation score, *EPDS* Edinburgh Postnatal Depression Scale, *STAI* State-Trait Anxiety Inventory, *Ref* Reference category, *CI* Confidence intervals, *GCSE* General Certificate of Secondary Education, *A level* Advanced Level.. ^a^ At any point in pregnancy; *N*=179

**Table 4:** Adjusted multivariate binary logistic regression identifying significant risk factors for excessive compared to normal GWG

	*P*	Exp(B)	95% CI
BMI pre-pregnancy			
Underweight	.716	1.68	.10, 27.12
Normal	*ref*		
Overweight	**<.001**	4.16	1.94, 8.89
Obese	**.010**	4.20	1.41, 12.50
Highest education level			
Left before GCSE	.148	.32	.07, 1.49
GCSE & Vocational	.613	1.27	.50, 3.23
A-level	.577	1.39	.44, 4.42
University	*ref*		
Postgraduate	.829	.92	.41, 2.06
Family income			
< 18,000	**.043**	.24	.06, .96
18–25,000	.358	.54	.15, 2.00
25–43,000	**.002**	.25	.10, .61
> 43,000	*ref*		
Do not wish to say	.890	.92	.30, 2.81
Alcohol in pregnancy^a^			
No	*ref*		
Yes	**.005**	.37	.19, .74
Term EPDS score	**.019**	1.10	1.02, 1.18

*BMI* Body Mass Index, *CI* Confidence intervals, *GCSE* General Certificate of Secondary Education, *A level* Advanced Level, *Ref* Reference category

^a^ At any point in pregnancy

R^2^ = .18 to .25; *N*=216

## Discussion

This study aimed to investigate the risk factors of excessive GWG in a UK population. This was the first study to investigate GWG in the UK utilising a biopsychosocial approach. Within the cohort, 15.3% had inadequate GWG, 28.7% normal GWG and 56.0% excessive GWG. Regarding risk factors, increased depressive symptoms on the EPDS and an overweight and obese pre-pregnancy BMI were associated with increased risk of excessive compared to normal GWG. Conversely, an income of less than £18,000 and £25–43,000 and consuming alcohol during pregnancy were associated with decreased risk of excessive GWG.

An important finding of this research is the high prevalence of excessive weight gain of 56.0%. This is considerably greater than that identified in a recent extensive systematic review, which found a worldwide prevalence of excessive GWG of 47%, already worryingly high. Given the poor outcomes associated with weight gain above or below Institute of Medicine recommended guidance, this figure is a cause for concern. Current NICE guidelines within the UK state that weight gain in pregnancy should not be tracked as a matter of routine, instead recommending guidance early in pregnancy on healthy diet and physical activity rather than on healthy weight gain in general. When considering the study that concluded pregnant women in the UK lacked concern regarding GWG, partly due to unclear advice from health professionals (18), this appears to be an area that should be reviewed. Women in the UK may benefit from a revised approach towards GWG in the various levels of the NHS.

We previously reported a prevalence of 14.3% for depression symptoms in our GiW population [19], similar to other research of this type. In this study increased prenatal depression symptoms were associated with increased risk of excessive GWG. Studies on GWG do not generally incorporate mental health measures. Those that do often include only psychological stress rather than mental health conditions such as perinatal depression or anxiety. A recent study that did incorporate a measure of depression identified that increased depressive symptoms on the EPDS were associated with higher risk of excessive GWG, similar to our findings [28]. However, another study with a measure of depression at the three month booking appointment identified that higher symptoms of depression were a protective factor [12]. These findings highlight the importance of incorporating measures of mood symptoms when investigating GWG and suggest that the timing of mood symptoms may be relevant to the risk of excessive GWG. While the relationship requires further exploration, our findings strengthen the argument to screen women for mental health problems as standard early in pregnancy in obstetric services, as this is a potentially targetable modifiable factor. This screening recommendation is also particularly important as prenatal depression is also associated with poor offspring health outcomes [29] as well as poor maternal health outcomes, for example through the association between depression and alcohol use in pregnancy [30]. This finding of an influence of a mental health factor highlights the importance of employing an overarching biopsychosocial approach to investigating complex areas such as GWG.

Pre-pregnancy BMI category was also linked to GWG in our cohort. When considering IOM categories split by pre-pregnancy BMI, it is clear that participants in all BMI categories are gaining above IOM recommendations, with excessive GWG being the most prevalent category throughout. Additionally, regarding risk factors for excessive GWG, an overweight or obese pre-pregnancy BMI was strongly associated with increased risk of excessive compared to normal GWG. Although the confidence intervals suggest this data should be interpreted with caution, reassuringly these findings are similar to existing literature in countries other than the UK, which consistently suggest that this is a risk factor for excessive weight gain [12, 13, 31]. In light of these findings it is vital to ensure all pregnant women in the UK receive advice on weight management at the earliest possible opportunity in pregnancy.

Consuming alcohol at any point in pregnancy was unexpectedly identified as a factor associated with a reduced risk of excessive weight gain. This is not a factor that has been found to be related to GWG in previous research and is challenging to interpret. Women who completely abstain from alcohol may be substituting with higher calorie non-alcoholic drinks. Alternatively, women drinking alcohol may be attempting to compensate for the alcohol by employing other improved health behaviours compared to those who do not drink alcohol [32], thus lowering the risk of excessive GWG. A third possibility is that alcohol is a proxy for another biopsychosocial factor not included in the model. This is important as the model adjusts for a number of factors, such as income and mental health, which are often linked to alcohol intake. Effectively, this approach may identify women who, in most respects, are undertaking healthy lifestyles but who very occasionally consume alcohol [32]. Finally, both an income of less than £18,000 and between £25–43,000 compared to the highest income category were found to decrease the risk of excessive weight gain. Although not a direct comparison to income, this contrasts with studies that suggested lower socioeconomic status [3, 13, 33] increased the risk of excessive weight gain. These findings require further exploration.

A limitation of the current study is that the cohort is based in Cardiff, South Wales and as such some of the findings may not be representative of other areas of the UK, or other nationalities. Additionally, our cohort recruited women who were booked specifically for an ELCS. Given the suggested relationship between GWG and increased risk of CS delivery (4), this could have influenced our findings. However, there was no significant differences in GWG between the various indications for ELCS which reduces the risk of this influence. Due to missing data in the regression analyses, the participant number for the unadjusted model was relatively low. It is possible this has led to overfitting of the initial model. However, as this was not an issue for our final adjusted models, we feel the impact on our findings is minimised. Whilst our study incorporated data from midwife reported medical notes, the questionnaire, including the pre-pregnancy weight utilised for GWG calculations, was completed by participant self-report which, although necessary, does have inherent potential biases to consider. Despite these noted limitations, this research offers important insight into GWG in the UK.

## Conclusion

This study identified a concerningly high prevalence of excessive GWG in our UK population, with a range of influencing factors. GWG is complex, and employing a biopsychosocial model provides a more overarching approach to identifying contributing factors. This research provides evidence for potential targets for future interventions to improve GWG outcomes. Moreover, given the poor outcomes associated with GWG, women in the UK may benefit from a revised approach towards GWG within the NHS, such as updated NICE guidelines to encourage tracking weight gain throughout pregnancy.

## Supplementary Information

**Additional file 1:.** Grown in Wales Prenatal Participant Questionnaire

## Data Availability

For this specific study, there is a danger that sharing participant data might reveal participant identity due to the nature of the data, the specific recruitment dates and unique route of recruitment. The datasets used and analysed during the current study will therefore not be made publically available but will be available from the corresponding author on reasonable request.

## Abbreviations

A level: Advanced Level
BMI: Body mass index
CBWC: Custom birthweight centiles
CI: Confidence intervals
CS: Caesarean section
ELCS: Elective caesarean section
EPDS: Edinburgh Postnatal Depression Scale
GCSE: General Certificate of Secondary Education
GiW: Grown in Wales
GWG: Gestational weight gain
IOM: Institute of Medicine
LGA: Large-for-gestational age infants
MRC: Medical Research Council
NHS: National Health Service
NICE: National Institute for Health and Care Excellence
REC: Research Ethics Committee
STAI: State-Trait Anxiety Inventory
UK: United Kingdom
WHO: World Health Organisation
WIMD: Welsh Index of Multiple Deprivation
