# Supplementary material for: Risk factors for excessive gestational weight gain in a UK population: a biopsychosocial model approach
Source: BMC Pregnancy Childbirth. 2021 Jan 10;21:43. doi: 10.1186/s12884-020-03519-1 (PMC7798251; doi:10.1186/s12884-020-03519-1)
Supplement: Supplementary file 1 — Additional file 1:. Grown in Wales Prenatal Participant Questionnaire [file 12884_2020_3519_MOESM1_ESM.pdf]

| Study ID: |
|---|
| Date: |

**Prenatal Participant Questionnaire**

**Title of Project: Grown in Wales**

**Version 1.1**

**22.05.15**

Thank you for answering these questions. It would help us if you could answer all the questions, however if you feel unhappy about answering any of them, please feel free to leave them blank. All information is kept in the strictest confidence and will not affect the standard of care you receive.

## QUESTIONNAIRE PART 1

Where known, please provide us with the answers to the following questions:

### About You

- **To which of these ethnic groups do you consider you belong?** *(Please tick)*

| | | | |
|---|---|---|---|
| Caucasian (White) | ☐ | Indian/Pakistani/Bangladeshi | ☐ |
| Far Eastern | ☐ | African/Afro-Caribbean | ☐ |
| South American/Hispanic | ☐ | Middle Eastern | ☐ |
| Other | ☐ | Do not wish to say | ☐ |

- **What is your country of birth?** *(Please tick)*

| | | | |
|---|---|---|---|
| Wales | ☐ | Scotland | ☐ |
| England | ☐ | Ireland | ☐ |
| Other *(please specify)* | ☐ | ……………………………… | |

- **What is your age?** …………..........

- **What was your weight before pregnancy?** ………..... **kg** **or** ……... **st** ......... **lbs**

- **What is <u>your</u> height? ………... m <u>or</u> ………... ft**

- **If you are towards term (37 weeks), please record your final weight:**

  **………... kg <u>or</u> …….. st ......... lbs at ………….. weeks**

- **Do you know <u>your</u> birthweight?** *(Please tick)*

  Yes ☐ it was …………………..

  No ☐

- **What is <u>your</u> highest level of education?** *(Please tick)*

| | | | |
|---|---|---|---|
| Left before GCSE | ☐ | GCSE/O levels | ☐ |
| A levels | ☐ | University | ☐ |
| Postgraduate Education | ☐ | Vocational Training | ☐ |

## <u>About your family</u>

- **To which of these ethnic groups does your baby's father belong?** *(Please tick)*

| | | | |
|---|---|---|---|
| Caucasian (White) | ☐ | Indian/Pakistani/Bangladeshi | ☐ |
| Far Eastern | ☐ | African/Afro-Caribbean | ☐ |
| South American/Hispanic | ☐ | Middle Eastern | ☐ |
| Other | ☐ | Do not wish to say | ☐ |

- **What is your <u>baby's father's</u> country of birth?** *(Please tick)*

| | | | |
|---|---|---|---|
| Wales | ☐ | Scotland | ☐ |
| England | ☐ | Ireland | ☐ |
| Other *(please specify)* | ☐ | ……………………………… | |

- **What is your family income per year before deductions?** *(Please tick)*

<£18,000 ☐ £18,000 - £25,000 ☐ £25,000 - £43,000 ☐

>£43,000 ☐ Do not wish to say ☐

**<u>About your pregnancy</u>**

- **How many weeks pregnant are you today?** ……….........

- **Is this pregnancy a natural or assisted conception?** *(Please tick)*

Natural ☐ Assisted *(IVF)* ☐ Do not wish to say ☐

- **Did you smoke in the three months <u>before</u> you found out you were pregnant?**

Yes (daily) ☐ Yes (Occasionally) but not every day) ☐

No ☐

- **Did you smoke <u>during</u> the first 12 weeks of your pregnancy?**:

Yes (daily) ☐ Yes (Occasionally) but not every day) ☐

No ☐

- **Did you smoke <u>after</u> the first 12 weeks of your pregnancy?**:

Yes (daily) ☐ Yes (Occasionally) but not every day) ☐

No ☐

- **Did you drink alcohol in the three months <u>before</u> you found out you were pregnant?**

Yes, almost every day ☐ Yes, once or twice a week ☐

Yes, once or twice a month ☐ Yes, once every couple of months ☐

No, ever ☐

- **Did you drink alcohol <u>during</u> the first 12 weeks of your pregnancy?**

  Yes, almost every day ☐ Yes, once or twice a week ☐

  Yes, once or twice a month ☐ Yes, once every couple of months ☐

  No, ever ☐

- **Did you drink alcohol <u>after</u> the first 12 weeks of your pregnancy?**

  Yes, almost every day ☐ Yes, once or twice a week ☐

  Yes, once or twice a month ☐ Yes, once every couple of months ☐

  No, ever ☐

- **Have you taken any illicit drugs during your pregnancy? Yes / No**

  If yes, what kind of drugs did you take? ……………………………………………

- **Have you done any strenuous exercise during your pregnancy? Yes / No**

  *(Strenuous exercise could be for example: running, jogging, squash, swimming, aerobics, cycling or football done for at least 30 minutes, at least once a week)*

- **Have you previously suffered from a diagnosed mood disorder (e.g. depression, bipolar disorder, pre or postnatal depression)?** *(Please tick)*

  Yes ☐ No ☐ Do not wish to say ☐

  **<u>If Yes:</u>**

  **Which Mood Disorder?...............................................................................................**

  **When was this first diagnosed (e.g. childhood, pre/during pregnancy)?**

  **…………………………………………………………………………………………………..**

  **Are you currently on medication for this mood disorder? Yes / No**

  *Please Specify which medication and when in pregnancy this was taken*

  ………………………………………………..................................................................

QUESTIONNAIRE PART TWO

DIRECTIONS: **<u>After</u> you found out you were pregnant, how often did you eat the following foods?** For <u>each</u> food type, please tick <u>one</u> box that best describes how often you ate this food. If this is different to before you were pregnant, please circle in the right hand column whether you ate more or less of this food after you found out you were pregnant. If this was not different, please leave the right hand column blank.

*Example: If since finding out you were pregnant you ate more chocolate, usually around 2 times per week, you would complete the row as shown:*

| | More than once/day | Once /day | 2-3 times per/week | Once in 2 weeks | Never/ Rarely | Since I found out I was pregnant I ate.. |
|---|---|---|---|---|---|---|
| Chocolate | | | ✔ | | | (More) / Less |

## Fruit and Vegetables

| | More than once/day | Once/ day | 2-3 times per/week | Once in 2 weeks | Never/ Rarely | Since I found out I was pregnant I ate.. |
|---|---|---|---|---|---|---|
| **Fresh Fruit** | | | | | | More / Less |
| **Dried fruit** e.g. raisins, dried apricots | | | | | | More / Less |
| **Salad and Cooked vegetables** | | | | | | More / Less |

## Dairy and Carbohydrates

| | More than once/day | Once /day | 2-3 times per/week | Once in 2 weeks | Never/ Rarely | Since I found out I was pregnant I ate.. |
|---|---|---|---|---|---|---|
| **Milk** | | | | | | More / Less |
| **Cheese, yoghurt** | | | | | | More / Less |
| **Bread, cereals, potatoes, rice, pasta** | | | | | | More / Less |

## Meat, Meat alternatives and Fish

| | More than once/day | Once /day | 2-3 times per/week | Once in 2 weeks | Never/ Rarely | Since I found out I was pregnant I ate.. |
|---|---|---|---|---|---|---|
| **Meat alternatives** e.g. beans, peas, tofu, soy | | | | | | More / Less |
| **Meat (unprocessed)** e.g. steak, ham, chicken slices | | | | | | More / Less |
| **Meat (processed)** e.g. Burgers, sausages, fried chicken | | | | | | More / Less |
| **Fish/shellfish** | | | | | | More / Less |

## Snacks

| | More than once/day | Once /day | 2-3 times per/week | Once in 2 weeks | Never/ Rarely | Since I found out I was pregnant I ate.. |
|---|---|---|---|---|---|---|
| **Chocolate** | | | | | | More / Less |
| **Chips, Crisps** | | | | | | More / Less |
| **Cakes, biscuits, ice cream** | | | | | | More / Less |
| **Take away meals** e.g. Chinese food, Curry | | | | | | More / Less |

## Drinks and Supplements

| | More than once/day | Once /day | 2-3 times per/week | Once in 2 weeks | Never/ Rarely | Since I found out I was pregnant I ate.. |
|---|---|---|---|---|---|---|
| **Soft drinks** | | | | | | More / Less |
| **Caffeine** e.g. tea, coffee, power drinks | | | | | | More / Less |
| **Supplements** e.g. iron, folate | | | | | | More / Less |

**QUESTIONNAIRE PART THREE**

DIRECTIONS: Please UNDERLINE the answer which comes closest to how you have felt in the **past week**, not just how you feel today. Here is an example already completed:

I have felt happy:

Yes, all the time

Y<u>es, most of the time</u>

No, not very often

No, not at all

This would mean: I have felt happy most of the time in the past few days.

Please complete the other questions in the same way. Do not take too long over it and make sure you answer all the questions.

**<u>IN THE PAST WEEK</u>**

1. I have been able to laugh and see the funny side of things:

As much as I always could

Not quite so much now

Definitely not so much now

Not at all

2. I have looked forward with enjoyment to things

As much as I ever did

Rather less than I used to

Definitely less than I used to

Hardly at all

3. I have blamed myself unnecessarily when things went wrong

Yes, most of the time

Yes, some of the time

Not very often

No, never

4. I have been anxious or worried for no good reason

    No, not at all

    Hardly ever

    Yes, sometimes

    Yes, very often

5. I have felt scared or panicky for no very good reason

    Yes, quite a lot

    Yes, sometimes

    No, not much

    No, not at all

6. Things have been getting on top of me

    Yes, most of the time I haven't been able to cope at all

    Yes, sometimes I haven't been coping as well as usual

    No, most of the time I have coped quite well

    No, I have been coping as well as ever

7. I have been so unhappy that I have had difficulty sleeping

    Yes, most of the time

    Yes, sometimes

    Not very often

    No, not at all

8. I have felt sad or miserable

    Yes, most of the time

    Yes, quite often

    Not very often

    No, not at all

9. I have been so unhappy that I have been crying

Yes, most of the time

Yes, quite often

Only occasionally

No, never

10. The thought of harming myself had occurred to me

Yes, quite often

Sometimes

Hardly ever

Never

DIRECTIONS: A number of statements which people have used to describe themselves are given below. Read each statement and then tick in the appropriate box on the right to indicate how you **generally** feel. There are no right or wrong answers. Do not spend too much time on any one statement but give the answer which seems to describe how you **generally** feel, even before pregnancy.

| | **In general….** | Almost never | Sometimes | Often | Almost always |
|---|---|---|---|---|---|
| 1. | I feel pleasant | | | | |
| 2. | I feel nervous and restless | | | | |
| 3. | I feel satisfied with myself | | | | |
| 4. | I wish I could be as happy as others seem to be | | | | |
| 5. | I feel like a failure | | | | |
| 6. | I feel rested | | | | |
| 7. | I am "calm, cool and collected" | | | | |

| | In general…. | Almost never | Sometimes | Often | Almost always |
|---|---|---|---|---|---|
| 8. | I feel that difficulties are piling up so that I cannot overcome them | | | | |
| 9. | I worry too much over something that really doesn't matter | | | | |
| 10. | I am happy | | | | |
| 11. | I have disturbing thoughts | | | | |
| 12. | I lack self-confidence | | | | |
| 13. | I feel secure | | | | |
| 14. | I make decisions easily | | | | |
| 15. | I feel inadequate | | | | |
| 16. | I am content | | | | |
| 17. | Some unimportant thought runs through my mind and bothers me | | | | |
| 18. | I take disappointments so keenly that I can't put them out of my mind | | | | |
| 19. | I am a steady person | | | | |
| 20. | I get in a state of tension or turmoil as I think over my recent concerns and interests | | | | |

Thank you for answering these questions. It would help us if you could answer all the questions, however if you feel unhappy about answering any of them, please feel free to leave them blank. All information is kept in the strictest confidence and will not affect the standard of care you receive.
